# Supplementary material for: Paleogenomes Reveal a Complex Evolutionary History of Late Pleistocene Bison in Northeastern China
Source: Genes (Basel). 2022 Sep 20;13(10):1684. doi: 10.3390/genes13101684 (PMC9602171; doi:10.3390/genes13101684)
Supplement: Supplementary file 1 [file genes-13-01684-s001.zip › genes-1895936-supplementary-done.pdf]

genes

Supporting Information

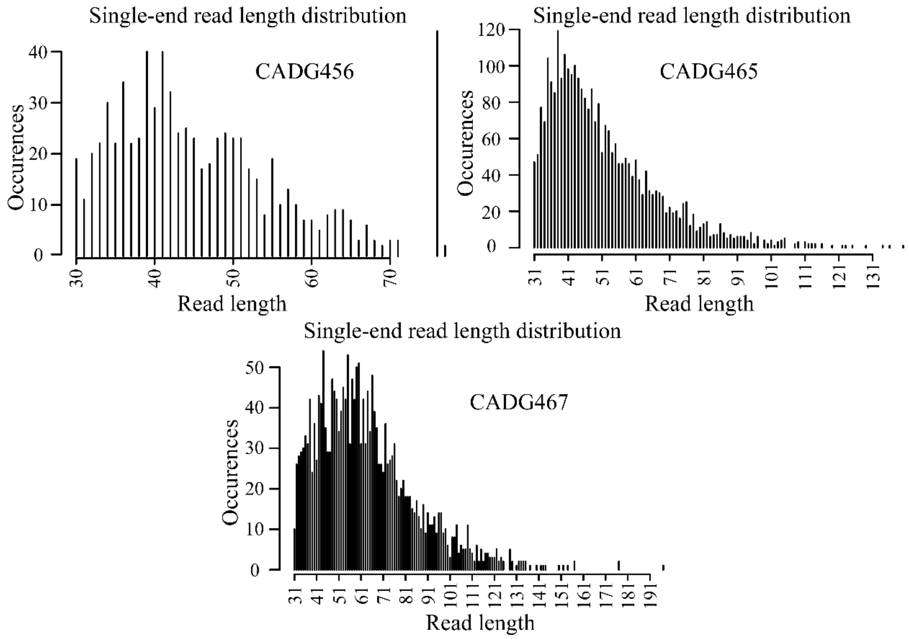

Figure S1: Estimated mitochondrial endogenous fragment length distributions for the 3 bison samples analyzed in this study.

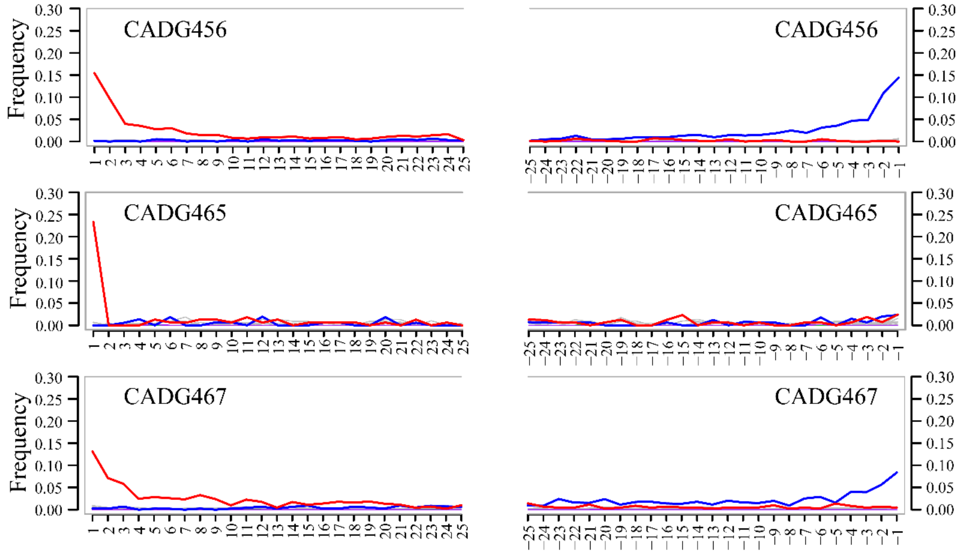

Figure S2: Mitochondrial cytosine deamination frequency inferred from the 3 bison samples analyzed in this study. *X axis* represents position from 5' (left) and 3' (right) read ends. Red line corresponds to C to T substitutions, blue line to G to A substitutions. Double stranded libraries were constructed from CADG456 and CADG465, while a single stranded library was obtained from CADG465.

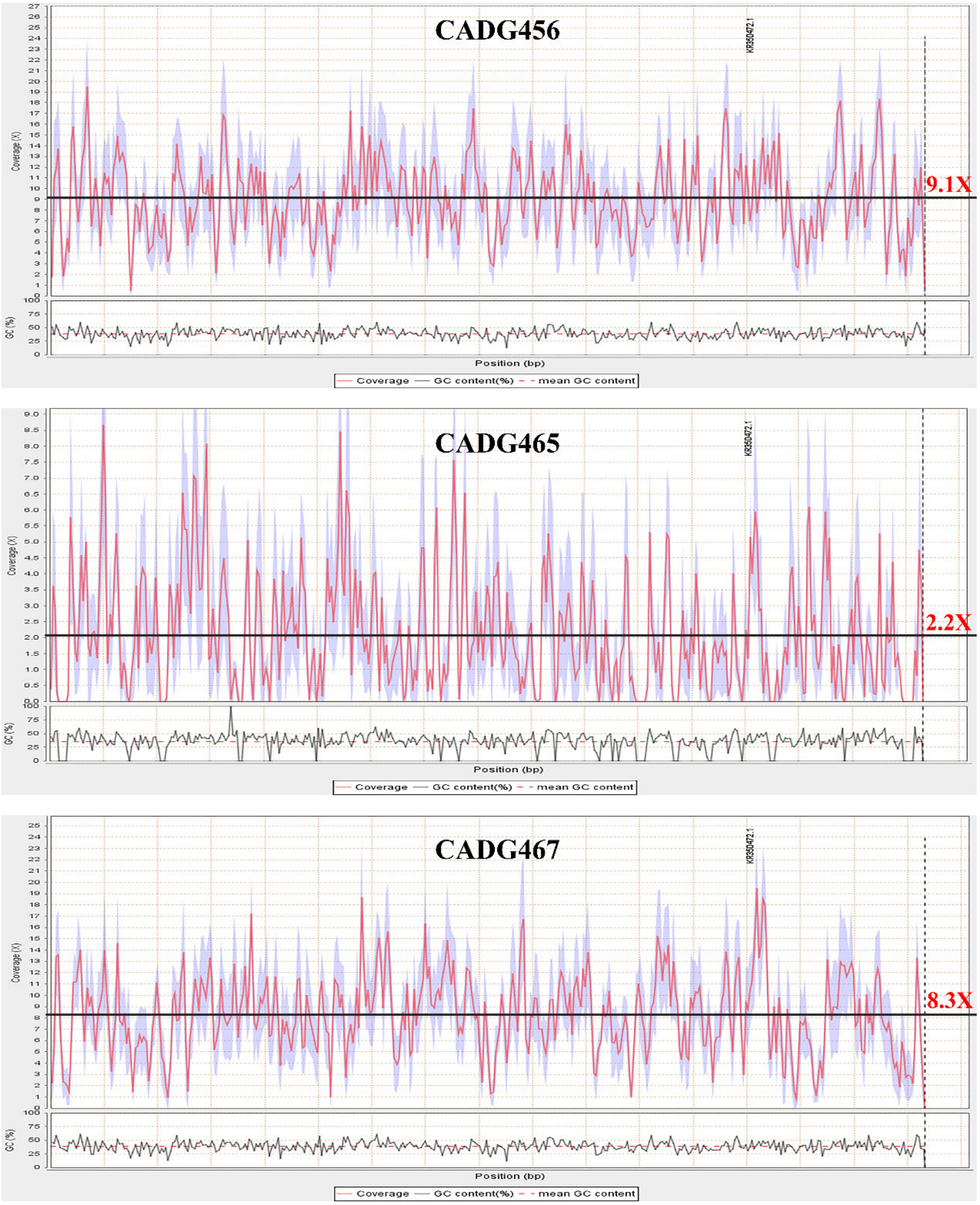

Figure S3: Coverage plots of the mitochondrial genomes obtained in this study. The three black lines represent the mean coverage of the three mitochondrial genomes.

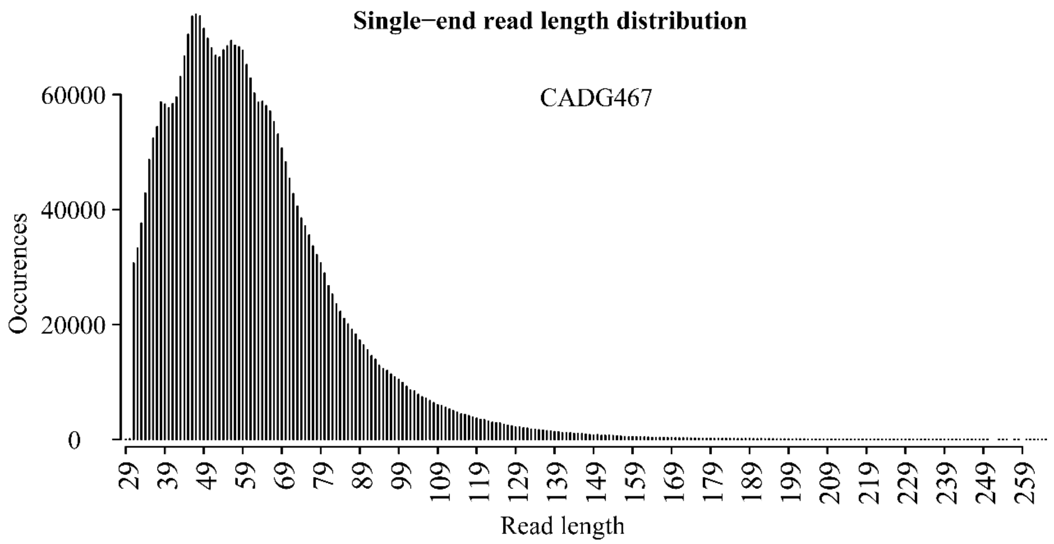

Figure S4: Estimated nuclear endogenous fragment length distributions for bison sample CADG467.

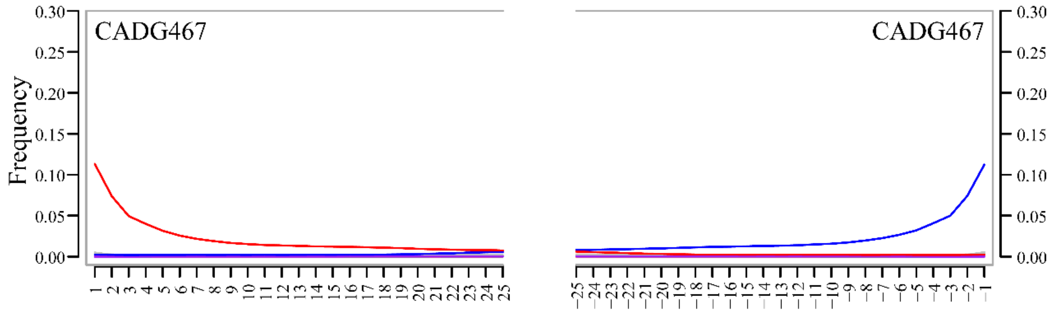

Figure S5: Nuclear cytosine deamination frequency inferred from bison sample CADG467. *X axis* represents position from 5' (left) and 3' (right) read ends. Red line corresponds to C to T substitutions, blue line to G to A substitutions.

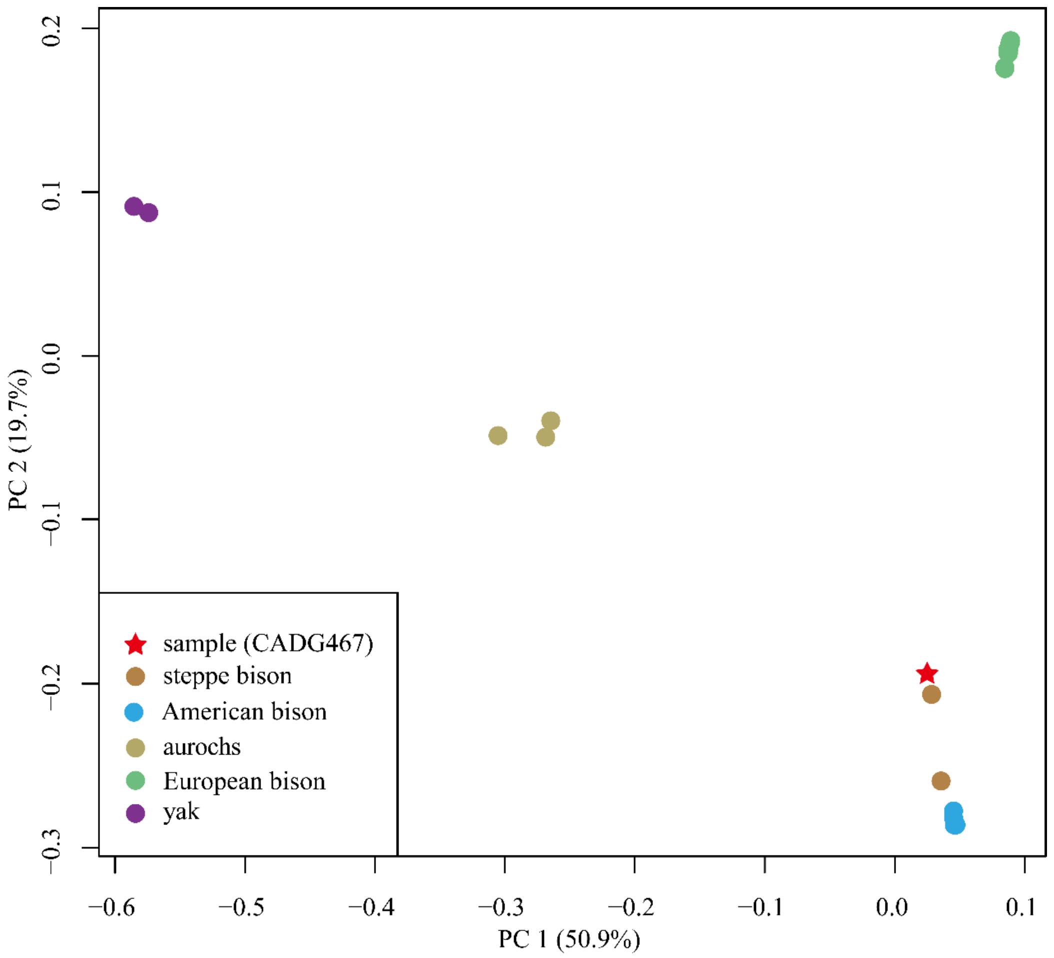

Figure S6: Principal component analysis (PCA) computed using genotype likelihoods with sample CADG467, two steppe bison, two yak, three aurochs, six American bison and ten European bison based on 6,000,104 SNPs.

Table S1: The information and mitochondrial mapping results of bison samples analyzed in this study.

**Sample No.**	**Haplogroup**	**Skeletal element**	**Location**	**Calibrated radiocarbon age (cal BP) and lab number**	**Library layout**	**mitogenome reference (GenBank No. KR350472)**				
						**Mapped reads**	**Mapped (bp)**	**Length (bp)**	**Average read depth (×)**	**Average fragment lengths (bp)**
CADG324	—	teeth	Dalian, Liaoning, China	—	paired	—	—	—	—	
CADG329	—	metacarpal bone	Dalian, Liaoning, China	—	paired	—	—	—	—	
CADG378	—	teeth	Zhaodong, Heilongjiang, China	—	paired	—	—	—	—	
CADG382	—	teeth	Zhaodong, Heilongjiang, China	—	paired	—	—	—	—	
CADG454	—	skull	Harbin, Heilongjiang	—	paired	—	—	—	—	
CADG455	—	skull	Harbin, Heilongjiang	—	paired	—	—	—	—	

CADG456	C	rib	Harbin, Heilongjiang	>43500 (Beta-609039)	paired	2896	148336	16120	9.087	51.22
CADG465	B	skull	Harbin, Heilongjiang	—	single-hybridization capture	754	35213	8871	2.1571	46.7
CADG467	C	skull	Harbin, Heilongjiang	34989-34228 (Beta-583723)	paired	2128	134886	15805	8.263	63.38
CADG468	—	skull	Harbin, Heilongjiang	—	paired	—	—	—	—	
CADG471	C	skull	Harbin, Heilongjiang	—	single-hybridization capture	288	16181	4138	0.9912	56.17
CADG519	—	metacarpal bone	Daqing, Heilongjiang	—	paired	—	—	—	—	
CADG536	—	radius	Daqing, Heilongjiang	—	paired	—	—	—	—	
CADG544	—	teeth	Qinggang, Heilongjiang	—	paired	—	—	—	—	
CADG605	C	-	Zhaodong, Heilongjiang	—	paired	111	6232	933	0.3818	56.14

CADG627	C	teeth	Qinggang, Heilongjiang	—	paired	101	5769	1231	0.3534	57.12
CADG654	—	astragalus	Zhaoyuan, Heilongjiang	—	paired	—	—	—	—	
CADG660	—	astragalus	Zhaoyuan, Heilongjiang	—	paired	—	—	—	—	
CADG687	—	teeth	Harbin, Heilongjiang	—	paired	—	—	—	—	
CADG711	—	teeth	Harbin, Heilongjiang	—	paired	—	—	—	—	
CADG714	—	teeth	Harbin, Heilongjiang	—	paired	—	—	—	—	

Table S2: Information of the four overlapping long range PCR (LR-PCR) primer pairs used in hybridization capture library construction.

Primer Name	Length	Tm°C	GC%	Sequence (5′-3′)	PCR product length (bp)
696_For	20	59.5	55	AAGCTAACAGGAGTACGGCG	4921
5616_Rev	21	59.1	52.4	AGCCCTGTGGTGAGTTTACAC	
5024_For	20	61.6	60	CTCCCTCCCCTATCTGGGTT	4954
9977_Rev	18	62.1	66.7	GGAGAAGGGAAGACGGGC	
9161_For	23	61.6	47.8	AGCACCTTCCAAGGACATCATAC	5049
14209_Rev	22	61.5	50	AGCAGTACCCTGAGATTTGGCT	
13229_For	22	62.3	54.5	GTTGGCAGTCTCGCACTAACAG	3979
883_Rev	24	62.3	50	GTGTTTAGGGCTAAGCATAGTGGG	

Table S3: Mapping results against different bison mitochondrial genomes as reference.

**Sample No.**	**Haplogroup**	**mitogenome reference**							
		**KR350472**		**KM593920**		**KX269145**		**GU946980**	
		**Length (bp)**	**Average read depth (×)**	**Length bp**	**Average read depth (×)**	**Length bp**		**Average read depth (×)**	
CADG456	C	16120	9.087	15896	8.765	15890	8.7368	15801	8.6881
CADG465	B	8871	2.1571	8933	2.1473	8876	2.169	8932	2.1591
CADG467	C	15805	8.263	15680	8.087	15467	7.9639	15515	7.9786
CADG471	C	4138	0.9912	4068	0.9651	3392	0.9588	4022	0.9574
CADG605	C	933	0.3818	931	0.3716	852	0.359	895	0.3629
CADG627	C	1231	0.3534	1231	0.3483	1179	0.3473	1191	0.3458

Table S4: Information of the nuclear genomes used in this study.

Species	N	Reference	Sample No	Run	Mapped reads	Mapped bp	Average read depth (×)	Average fragment lengths (bp)
		In this study	CADG467	—	3,274,372	201,224,410	0.0759	61.46
	2	(Richards et al., 2019a, 2019b)	875	SRR8561392	6,425,886	565,312,668	0.2132	87.98
				SRR8561399				
			3133	SRR8561407	8,710,514	799,684,687	0.3016	91.81
				SRR8561397				
				SRR10247001				
Bison bison	6	(Wu et al., 2018), PRJNA748091, PRJNA658430	bisonmzc	SRR6448740	499,505,142	63,723,062,065	24.032	127.96
				SRR6448738				
				SRR6448739				
				SRR6448737				
			P8	SRR12514560	158,019,607	15,826,804,131	5.9688	100.31
			P9	SRR12514559	223,386,278	22,324,116,428	8.4191	100.06
			P10	SRR12514558	258,526,551	25,844,574,025	9.7468	100.09
			100490	SRR15221439	300,740,275	45,594,484,928	17.1951	152.33
			100491	SRR15221438	246,023,167	37,294,915,599	14.0651	152.24
Bos primigenius	3	(Verdugo et al. 2019)	ch22	ERR3317469-ERR3317482	9,740,305	549,080,961	0.2071	56.37
			gyu2	ERR3317392-ERR3317399	56,878,536	2,772,528,865	1.0456	48.75
			th7	ERR3317483-ERR3317503	2,365,117	106,412,027	0.0401	44.99
Bison bonasus	10	(Wu et al., 2018)	Winst1	SRR6448681	96,783,876	12,123,380,625	4.5721	125.49
			Winst2	SRR6448680	95,524,076	11,973,036,451	4.5154	125.59
				SRR6448675				
			Winst3	SRR6448672	104,983,658	13,164,952,996	4.9649	125.6
			Winst4	SRR6448677	96,448,886	12,119,126,405	4.5705	125.87
			Winst5	SRR6448676	101,626,482	12,750,662,720	4.8087	125.72
				SRR6448671				
			Winst8	SRR6448673	111,870,568	14,010,931,523	5.284	125.45
			Winst9	SRR6448685	102,037,272	12,759,688,476	4.8121	125.28
			Winst10	SRR6448674	114,761,924	14,364,154,785	5.4172	125.35
			Winst11	SRR6448684	749,141,984	93,760,756,138	35.3602	125.36
				SRR6448682				
				SRR6448670				
				SRR6448683				

			Winst12	SRR6448679	111,179,527	13,927,313,183	5.2524	125.47
Bos grunniens	2	(Qiu et al., 2015)	Dyg46	SRR2058191	155,776,185	15,891,797,979	5.9933	102.43
			Dyg66	SRR2058054	150,992,120	15,203,366,698	5.7337	101.58

Table S5: Complete mitochondrial genomes and ages of steppe bison, American bison and yak downloaded from NCBI.

**Species**	**NCBI Accession No.**	**Locality**	**Age (yr BP)**
steppe bison	KR350472	Rauchua River, Russia	10,817
steppe bison	KX269109	Chukotka, Russia	25,247
steppe bison	KX269110	Taimyr, Russia	/
steppe bison	KX269111	Taimyr, Russia	/
steppe bison	KX269112	Taimyr, Russia	47,452
steppe bison	KX269113	Taimyr, Russia	35,827
steppe bison	KX269114	Taimyr, Russia	36,296
steppe bison	KX269115	Taimyr, Russia	46,823
steppe bison	KX269116	Taimyr, Russia	38,227
steppe bison	KX269117	British Columbia, Canada	11,871
steppe bison	KX269118	British Columbia, Canada	11,598
steppe bison	KX269119	Alberta, Canada	12,927
steppe bison	KX269120	Alberta, Canada	/
steppe bison	KX269121	Alberta, Canada	13,165
steppe bison	KX269122	Colorado, USA	/
steppe bison	KX269123	Northwest Territories, Canada	13,686
steppe bison	KX269124	Northwest Territories, Canada	13,094
steppe bison	KX269125	Northwest Territories, Canada	11,314
steppe bison	KX269126	British Columbia, Canada	12,253
steppe bison	KX269127	Yukon, Canada	/
steppe bison	KX269128	Yukon, Canada	/
steppe bison	KX269129	Yukon, Canada	30,614
steppe bison	KX269130	Yukon, Canada	/
steppe bison	KX269131	Yukon, Canada	/
steppe bison	KX269132	Yukon, Canada	42,462
steppe bison	KX269133	Yukon, Canada	/
steppe bison	KX269134	Yukon, Canada	/
steppe bison	KX269135	Yukon, Canada	/

steppe bison	KX269136	Yukon, Canada	13,861
steppe bison	KX269137	Yukon, Canada	14,219
steppe bison	KX269138	Yukon, Canada	14,360
steppe bison	KX269139	Yukon, Canada	14,409
steppe bison	KX269140	Yukon, Canada	/
steppe bison	KX269141	Yukon, Canada	125,000
steppe bison	KX269142	Yukon, Canada	/
steppe bison	KX269143	Alberta, Canada	4,518
steppe bison	KX269144	Alberta, Canada	13,008
steppe bison	KX269145	Alberta, Canada	13,023
steppe bison	KM593920	France	15,880
steppe bison	MN549280	Alaska, USA	59,648
steppe bison	MN049905	Maly Anuy River, Russia	/
steppe bison	MN049906	Maly Anuy River, Russia	/
steppe bison	MN049907	Maly Anuy River, Russia	/
steppe bison	MN049908	East Siberia, Russia	/
steppe bison	MN049909	Maly Anuy River, Russia	/
steppe bison	MN049910	East Siberia, Russia	/
steppe bison	MN049911	Rauchua River, Russia	/
steppe bison	MN049912	Ostrovnovskaya tundra, Russia	/
steppe bison	MN049913	East Siberia, Russia	/
steppe bison	NC_027233	France	/
steppe bison	MF134653	Yukon, Canada	/
steppe bison	KX592174	Yukon, Canada	/
steppe bison	KX898010	France	17,450
steppe bison	KX898014	France	37,700
steppe bison	KX898018	East Siberia, Russia	27,800
steppe bison	KX898019	East Siberia, Russia	12,927
steppe bison	KX898020	East Siberia, Russia	/
steppe bison	3133	Yukon, Canada	26,360
steppe bison	875	Siberia, Russia	/
American bison	GU946976	/	modern
American bison	GU946977	/	modern

American bison	GU946978	/	modern
American bison	GU946979	/	modern
American bison	GU946980	/	modern
American bison	GU946981	/	modern
American bison	GU946982	/	modern
American bison	GU946983	/	modern
American bison	GU946984	/	modern
American bison	EU177871	/	modern
Yak	KM233416	/	/
Yak	KJ704989	/	/
Yak	AY684273	/	/
Yak	KJ463418	/	/

Table S6: D-loop sequences of steppe bison used in this study.

Location	Sample
North America south of Beringia and Eastern Beringia	BS99, BS100, BS102, BS107, BS129, BS162, BS173, BS342, BS368, BS417, BS421, BS422, BS423, BS426, BS432, BS433, BS434, BS439, BS440, BS443, BS444, BS454, BS464, BS465, BS466, BS560, BS569, BS605
Eastern Beringia	BS111, BS150, BS165, BS172, BS176, BS211, BS222, BS236, BS244, BS260, BS284, BS311, BS414, BS478
China, Western Beringia and Eastern Beringia	BS105, BS133, BS233, BS243, BS282, BS286, BS320, BS328, BS388, BS396, BS397, BS405, BS415, BS418, BS459, BS495, BS498, BS564, BS571
North America south of Beringia and Eastern Beringia	BS108, BS109, BS121, BS123, BS124, BS125, BS147, BS161, BS163, BS164, BS178, BS193, BS196, BS198, BS202, BS237, BS249, BS297, BS359, BS387, BS392, BS393, BS394, BS395, BS412, BS469, BS472, BS473, BS477, BS497, BS499, BS500, BS503, BS517

Due to the poor DNA preservation, we only retrieved partial mitochondrial genomes from three samples (i.e., CADG471, CADG605 and CADG627), hence they were not included in the phylogenetic reconstructions. Although the sequences show an average mitochondrial coverage below 1x, we were nonetheless able to genotype these individuals from the portions of the mitochondrial genome that were recovered. Firstly, using the available complete mitogenomes of haplogroups A, B and C as reference, we identified nucleotide substitutions that were unique to haplogroup C. Secondly, we manually inspected the three partial mitochondrial genomes (CADG471, CADG605 and CADG627), which were covered by a minimum of two reads, looking for informative substitutions. In these three samples, we totally identified 15 unique nucleotide substitutions sites which owned only by haplogroup C-specific substitutions. The results indicate that these three samples should all belong to the bison haplogroup C (Table S7).

Table S7: nucleotide substitutions in the mitochondrial genome that were unique to the individuals CADG456, CADG467 and the mitochondrial reference KR350472.

Haplogroup	Sample	Locus														
		7	6	8	8	8	9	9	9	1	1	1	1	1	1	1
		0	5	3	4	6	1	1	9	0	1	2	3	5	5	5
		1	3	4	3	4	4	8	0	7	1	5	7	0	3	5
			5	5	8	8	8	7	8	1	6	8	6	6	9	5
										6	6	0	5	9	9	8
A		C	T	C	G	C	G	T	T	C	C	A	T	T	C	C
B		C	T	C	G	C	G	T	T	C	C	A	T	T	C	C
C	KR350472	T	C	T	A	T	A	C	C	C	T	G	G	C	T	T
	CADG456	T	C	T	A	T	A	C	C	T	T	G	G	C	T	T
	CADG467	T	C	T	A	T	A	C	C	T	T	G	G	C	T	T
	CADG471	—		T	—	—	A	—	C	—	—	G	—	C	—	T
	CADG605	—	C	T	A	—	A	C	—	T	T	—	G	—	—	—
	CADG627	T	—	—	—	T	—	C	—	—	—	—	—	—	T	—

Table S8: Mitochondrial genomes of three steppe bison haplogroups.

Haplogroup	Location	Samples
A	North America	MN549280, KX269144, KX269131, KX269132, KX269124, KX269123, KX269119, KX269118, KX269126, KX269145, KX269135, KX269134, KX269127, MN049910, KX269141, MN049908, KX269125, KX269137, KX269139, KX269140, KX269136, KX269129, KX269117, KX269143, KX269121, KX269122, KX269142, KX269120, KX592174, MF134653, 3133
B	Russia, France	KX898010, KM593920, NC_027233, KX269109, MN049913, MN049912, MN049905, KX898019, KX269115, KX269112, KX898014, KX269130, KX269138,

		KX269111, KX269133, KX269110, KX898018, KX898020, KX269113, KX269114, KX269116, MN049906, MN049911, MN049909, MN049907, CADG465, 875
C	Russia, China	KR350472, CADG456, CADG467, CADG471, CADG605, CADG627

References

1. Richards, S.M.; Hovhannisyan, N.; Gilliham, M.; Ingram, J.; Skadhauge, B.; Heiniger, H.; Llamas, B.; Mitchell, K.J.; Meachen, J.; Fincher, G.B.; et al. Correction: Low-cost cross-taxon enrichment of mitochondrial DNA using in-house synthesised RNA probes. PloS one 2019, 14, e0213296, doi:10.1371/journal.pone.0213296.
2. Richards SM, M.K., Tobler R, Cooper A. Recombinase Polymerase Amplification (RPA) versus PCR for ancient DNA library amplification. PeerJ Preprints 2019, 7, 27544v27541,
3. Wu, D.D.; Ding, X.D.; Wang, S.; Wójcik, J.M.; Zhang, Y.; Tokarska, M.; Li, Y.; Wang, M.S.; Faruque, O.; Nielsen, R.; et al. Pervasive introgression facilitated domestication and adaptation in the Bos species complex. Nature ecology & evolution 2018, 2, 1139-1145, doi:10.1038/s41559-018-0562-y.
4. Verdugo, M.P.; Mullin, V.E.; Scheu, A.; Mattiangeli, V.; Daly, K.G.; Delser, P.M.; Hare, A.J.; Burger, J.; Collins, M.J.; Kehati, R.; et al. Ancient cattle genomics, origins, and rapid turnover in the Fertile Crescent. Science 2019, 365, 173-176,
5. Qiu, Q.; Wang, L.Z.; Wang, K.; Yang, Y.Z.; Ma, T.; Wang, Z.F.; Zhang, X.; Ni, Z.Q.; Hou, F.J.; Long, R.J.; et al. Yak whole-genome resequencing reveals domestication signatures and prehistoric population expansions. Nat Commun 2015, 6, 10283, doi:10.1038/ncomms10283.
